# Electroacupuncture alleviates Parkinson's disease-related pain by inhibiting microglial NLRP3-ASC inflammasome in the amygdala

**DOI:** 10.1590/1414-431X2026e15248

**Published:** 2026-07-10

**Authors:** Xiao-Wei Wang, Ben-Fan Zhu, Ying Shi, A-Long Xia, Ai-Ling Wang, Jing-Jing Peng, Si-Huan Zhu, Zhi-Lai Yang, Jun Li

**Affiliations:** 1 Department of Neurology, The First Affiliated Hospital of Anhui University of Chinese Medicine, Hefei, China; 2 Department of Pain, The First Affiliated Hospital of Anhui Medical University, Hefei, China; 3 Department of Anesthesiology, The First Affiliated Hospital of Anhui Medical University, Hefei, China

**Keywords:** Electroacupuncture, Parkinson's disease-related pain, Microglia, NLRP3, Amygdala, Neuroinflammation

## Abstract

This study aimed to investigate the role of microglia and NOD-like receptor protein 3 (NLRP3) inflammasome-mediated neuroimmune pathways in the analgesic effects of electroacupuncture (EA) in a mouse model of Parkinson's disease (PD). Male C57BL/6 mice (8 weeks old) were randomly assigned to the control, PD model, and PD + EA groups. PD was induced by intraperitoneal injection of 1-methyl-4-phenyl-1,2,3,6-tetrahydropyridine (MPTP), while control mice received saline. EA was administered to the motor cortex once daily for five consecutive days in the PD + EA group, whereas PD model mice were restrained without receiving EA stimulation. Behavioral assessments were performed to evaluate motor function and nociceptive sensitivity. Immunohistochemistry, immunofluorescence, and western blot analyses were used to quantify tyrosine hydroxylase (TH), ionized calcium-binding adapter molecule 1 (Iba-1), NLRP3 inflammasome components, and inflammatory cytokines in key brain regions. Compared with control mice, PD model mice showed reduced motor performance and heightened nociceptive sensitivity, accompanied by a decrease in TH-positive neurons and an increase in Iba-1-positive microglia in both the substantia nigra and amygdala. EA significantly improved motor performance and increased pain thresholds. Moreover, EA preserved TH-positive neurons, suppressed microglial activation, and downregulated the expression of NLRP3, ASC, caspase-1, interleukin (IL)-1β, IL-6, and tumor necrosis factor (TNF)-α in the amygdala. These findings suggest that EA alleviates PD-related pain, possibly by modulating microglial activation and NLRP3 inflammasome signaling in the amygdala, thereby reducing neuroinflammation.

## Introduction

Parkinson's disease (PD) is a progressive neurodegenerative disorder characterized by a wide range of motor and non-motor symptoms, including pain, all of which substantially impair patients’ quality of life (1). Key regions within the central nervous system involved in pain processing in PD include the amygdala, thalamus, prefrontal cortex, anterior cingulate cortex, and spinal cord (2). Among these, the amygdala, a critical component of the limbic system, plays a pivotal role in both nociceptive regulation and neuroinflammatory signaling (3). Although neuroinflammation has been known to contribute to the pathogenesis of PD-associated pain, the precise mechanisms linking this association remain incompletely understood (4). Thus, elucidating the neuroimmune mechanisms underlying PD-related pain is a priority for future investigation.

Microglia, the resident immune cells of the central nervous system, are rapidly activated in response to injury or pathological insults (5). Upon activation, microglia release pro-inflammatory cytokines, including interleukin-1β (IL-1β), interleukin-6 (IL-6), and tumor necrosis factor-α (TNF-α), which act on supraspinal structures involved in pain modulation (6). The NLRP3 inflammasome, a multiprotein complex, is a crucial component of the innate immune system and plays a key role in initiating inflammatory responses (7). It is widely expressed across various cell types, including neurons, dendritic cells, neutrophils, macrophages, and particularly microglia (8). Overactivation of the microglial NLRP3 inflammasome has been implicated in numerous neurodegenerative diseases, including PD (9).

Current pharmacological treatments for PD-associated pain include dopaminergic agents, γ-aminobutyric acid (GABA) analogs, and prolonged-release oxycodone/naloxone (OXN-PR) combination analgesics, while non-pharmacological approaches involve acupuncture, neuromodulation, exercise, and physiotherapy (10). However, the underlying therapeutic mechanisms of these interventions remain insufficiently characterized. Notably, repetitive transcranial magnetic stimulation (rTMS) targeting the primary motor cortex (M1) has demonstrated efficacy in alleviating PD-related musculoskeletal pain (11). Electroacupuncture (EA), a widely used neuroregulatory intervention in traditional Chinese medicine, has demonstrated efficacy in reducing neuropathic pain by modulating brain networks, neural activity, and neurochemical metabolism (12,13).

In this study, we explored the neuroimmune mechanisms underlying EA's alleviation of nociceptive hypersensitivity in 1-methyl-4-phenyl-1,2,3,6-tetrahydropyridine (MPTP)-induced PD mice. Specifically, we investigated the effects of EA on microglial activation and NLRP3 inflammasome signaling in the amygdala following EA stimulation over the motor area.

## Material and Methods

### Animals

A total of 27 SPF-grade male C57BL/6 mice (8 weeks old, 22±2 g) were obtained from Hangzhou Ziyuan Experimental Animal Technology Co., Ltd. (China). All mice were housed in a temperature-controlled environment (23±2°C) with 60% relative humidity and a 12-h light/dark cycle, with free access to food and water.

All experimental procedures were conducted in accordance with the National Institutes of Health Guide for the Care and Use of Laboratory Animals and were approved by the Animal Ethics Committee of Anhui University of Chinese Medicine (Approval No. AHUCM-mouse-2023001) and conducted in accordance with institutional and national guidelines.

### Experimental design

After a one-week acclimation period, the mice were randomly assigned to three groups: control, PD model, and PD model + EA (n=9 each group). A subacute PD model was established by intraperitoneal injection of MPTP (30 mg/kg per day; S47312, Selleck Chemicals, USA) for five consecutive days, according to previously established guidelines (14). Control mice received equivalent volumes of saline.

After modeling, mice in the PD and PD + EA groups were immobilized using a custom-designed head fixator (Patent No. CN202220053210.7). For EA treatment, sterile disposable acupuncture needles (0.25×25 mm) were inserted bilaterally into sites corresponding to the scalp over the motor area, approximately 3 mm lateral to the ear and oriented toward the contralateral eye (15). Stimulation parameters were set to a dense-disperse wave at 2/15 Hz, with an intensity of 0.8-1 mA, delivered for 20 min daily (16). Current intensity was adjusted to maintain calm behavior, allowing for mild ear twitching or muscle contractions. EA was administered once daily for five consecutive days. Mice in the PD model group underwent identical fixation procedures but received no EA stimulation. This design controlled for non-specific effects of handling and immobilization. Details of group assignments, treatments, and stimulation parameters are summarized in Table 1.

**Table 1:** Experimental groups and intervention parameters.

Group	n	Treatment	MPTP Dose	EA Parameters	Stimulation Duration
Control	9	Intraperitoneal injection of saline for 5 consecutive days	30 mg/kg	-	0
PD model	9	MPTP injection (5 days), then placed in head fixator. Bilateral scalp needle insertion over motor cortex (3 mm depth), without electrical stimulation, once daily for 5 days (20 min/session). Sterile acupuncture needles (0.25×0.25 mm) were used.	30 mg/kg (1 mg/mL)	Needle insertion only (no electrical current)	20 min/day × 5 days
PD model + EA	9	Same needle placement as sham group. EA applied at 2/15 Hz dense-disperse wave, 0.8-1 mA, for 20 min daily for 5 days.	30 mg/kg (1 mg/mL)	2/15 Hz, 0.8-1 mA, dense-disperse wave	20 min/day × 5 days

PD: Parkinson's disease; EA: electroacupuncture; MPTP: methyl-4-phenyl-1,2,3,6-tetrahydropyridine.

The substantia nigra (SN) and amygdala were identified based on anatomical landmarks and dissected under a stereomicroscope (CX41, Olympus Corp., Japan). Sampling accuracy was further verified according to stereotaxic coordinates from Paxinos and Franklin's The Mouse Brain in Stereotaxic Coordinates (17), with the SN located between bregma −2.80 and −3.40 mm and the amygdala between bregma −1.06 and −1.82 mm. Brain tissue was collected following decapitation under anesthesia. Bilateral amygdala and SN regions were dissected according to atlas-based coordinates. For immunohistochemistry, brains were paraffin-embedded and coronally sectioned at 6-8-μm thickness. Serial sections were collected at 6-8-μm intervals.

### Blinding procedures

All behavioral tests were conducted by investigators who were blinded to group assignments. Group allocations were coded and concealed from behavioral scorers and data analysis until all experiments were completed. EA treatments and MPTP modeling were performed by a separate investigator who was not involved in behavioral testing, immunohistochemical staining, or western blot analysis.

### Behavioral testing

*Open-field test*. Spontaneous locomotor activity was assessed using the open-field test and analyzed with the Super Maze 2.0 video tracking system (Shenzhen RWD Life Science Co., Ltd., China). To minimize external disturbances, no more than two investigators were present during each trial. Each mouse was gently placed in the center of the test arena in the same orientation, and locomotion was recorded for five minutes. Total distance traveled and average movement speed were quantified.

#### Pole test

Motor coordination and balance were evaluated using a pole test. A vertical wooden pole wrapped in gauze was used as the apparatus. Mice were placed head-up at the top of the pole, and the time required to turn and descend to the base was recorded as pole climbing time. Each mouse underwent three trials at three-minute intervals, and the average time was calculated.

#### Suspension test

Hindlimb grip strength was assessed using a custom-made hemp rope suspended 30 cm above the ground. Each mouse was gently placed on the rope, and hindlimb grasping was scored as follows: 3 points if both hindlimbs grasped the rope, 2 points if only one hindlimb grasped the rope, and 1 point if neither hindlimb maintained grip. This procedure was repeated five times per mouse, and the average score was calculated.

#### Von Frey test

Mechanical pain sensitivity was assessed using the Von Frey test. After acclimatization in the testing cages, a series of calibrated Von Frey monofilaments were applied perpendicularly to the plantar surface of the left hind paw. The mechanical withdrawal threshold was defined as the minimum force (in grams) required to elicit a brisk withdrawal response. Each mouse was tested three times with at least a 10-min interval between each trial, and the mean value was recorded for analysis.

#### Hot plate test

Thermal sensitivity was evaluated using the hot plate test. Mice were placed individually on a glass platform within a testing chamber and allowed to acclimate. An infrared heat source was then directed at the plantar surface of the left hind paw. The withdrawal latency was defined as the time (in seconds) from heat stimulus onset to the paw withdrawal, licking, or jumping. Each mouse underwent three trials with at least a 10-min interval between trials. A cutoff of 25 s was set to prevent tissue damage, and the average latency was recorded.

### Immunohistochemistry (IHC) analysis

Tyrosine hydroxylase (TH)-positive neurons in the SN and amygdala were detected by IHC. Mice were anesthetized and perfused transcardially, and the brains were extracted and post-fixed in 4% paraformaldehyde (PFA) for 1 h. Brain tissue samples were cryoprotected in 15, 20, and 30% sucrose solutions, embedded in OCT compound, and coronally sectioned. Sections were dried at 37°C and blocked in 5% bovine serum albumin (BSA) containing 0.5% Triton X-100 in PBS for 1 h at room temperature. Sections were incubated overnight at 4°C with monoclonal anti-TH antibody (1:500, Abcam, ab75875, USA), followed by PBS washes and incubation with fluorescently labeled secondary antibodies (1:500, Abcam, ab6721). Sections were counterstained with DAPI and visualized using a fluorescence microscope (CX41, Olympus Corp.). Quantification of TH-positive cells in the SN and amygdala was performed using ImageJ software (NIH, USA). Cell counts were obtained from three representative sections per animal in a consistent region of interest (ROI) across groups. Two trained investigators independently counted TH-positive neurons under blinded conditions, and the average of their counts was used for subsequent analysis.

### Immunofluorescent staining (IHF) analysis

Microglial activation in the amygdala was assessed by IHF detection of Iba-1. Brain cryosections were washed with PBS and treated twice with 0.6% hydrogen peroxide for 15 min each. Sections were then permeabilized in 0.4% Triton X-100 in TBS for 1 h at room temperature and blocked in 5% goat serum for 30 min. Primary antibodies were applied and incubated for 40 h at 4°C in a humidified chamber (anti-Iba1 antibody, 1:1200, Abcam, ab178846). After PBS rinsing, sections were incubated with fluorescent secondary antibodies (Alexa Fluor^®^ 488, 1:1000, Abcam, ab150081) in the dark for 90 min at 37°C, counterstained with DAPI, washed, mounted with antifade mounting medium, and imaged with the fluorescence microscope.

### Western blot analysis

Expression levels of inflammatory mediators, including NLRP3, ASC, caspase-1, IL-6, IL-1β, and TNF-α, were assessed by using western blot (WB) analysis. SN and amygdala tissues were collected, snap-frozen in dry ice, and stored at −80°C. For each mouse, the brain was dissected, and part of the tissue was fixed for IHC/IHF analysis, while the remaining portion was preserved for protein extraction and WB analysis. Protein concentrations were determined using a bicinchoninic acid (BCA) assay kit (Beyotime, China). Total protein was extracted, separated by SDS‐polyacrylamide gel electrophoresis (SDS-PAGE), and transferred onto polyvinylidene difluoride (PVDF) membranes. After blocking, membranes were incubated overnight at 4°C with primary antibodies, including anti-NLRP3 antibody (1:2000, Proteintech, 30109-1-AP, China), anti-ASC antibody (1:5000, Proteintech, 10500-1-AP), anti-caspase-1 antibody (1:1000, Proteintech, 31020-1-AP), anti-IL-1β antibody (1:1000, Proteintech, 26048-1-AP), anti-IL-6 antibody (1:1000, Proteintech, 21865-1-AP), anti-TNF-α antibody (1:1000, Proteintech, 17590-1-AP), and anti-GAPDH antibody (1:5000, Proteintech, 10494-1-AP). After washing in Tris-Buffered Saline (TBS) and Tween 20 (TBST) for 8 min at room temperature, membranes were incubated with HRP-conjugated secondary antibodies (1:2000, Proteintech, SA00001-2) for 1 h with gentle agitation. Then, blots were washed twice with TBST, and once in TBS for 8 min, followed by incubation with chemiluminescent substrate for 3 min. Blots were visualized using a Bio-Pro gel imaging system (Tanon, China). Band densities were quantified using Quantity One software (Tanon).

### Statistical analysis

Statistical analysis was performed using SPSS version 25.0 (IBM, USA). Data are reported as means±SD. Sample sizes were based on experimental feasibility and consistency with previously reported MPTP protocols using similar outcome measures. Normality and homogeneity of variance were assessed prior to analysis. Group comparisons were conducted using independent sample *t*-tests. A P-value <0.05 was considered statistically significant.

## Results

### EA improved motor function in MPTP-induced mice

MPTP-induced PD mice showed significant motor deficits compared to control mice, including a reduced total distance traveled (15.89±4.58 m *vs* 11.00±2.71 m, *t*=2.598, P=0.019), slower average speed (0.05±0.02 m/s *vs* 0.04±0.012 m/s, *t*=2.616, P=0.019), longer pole climbing time (3.27±0.44 s *vs* 6.61±1.07 s, *t*=−8.140, P<0.0001), and lower suspension scores (2.56±0.44 *vs* 1.59±0.31, *t*=5.049, P<0.0001).

**Figure 1:**
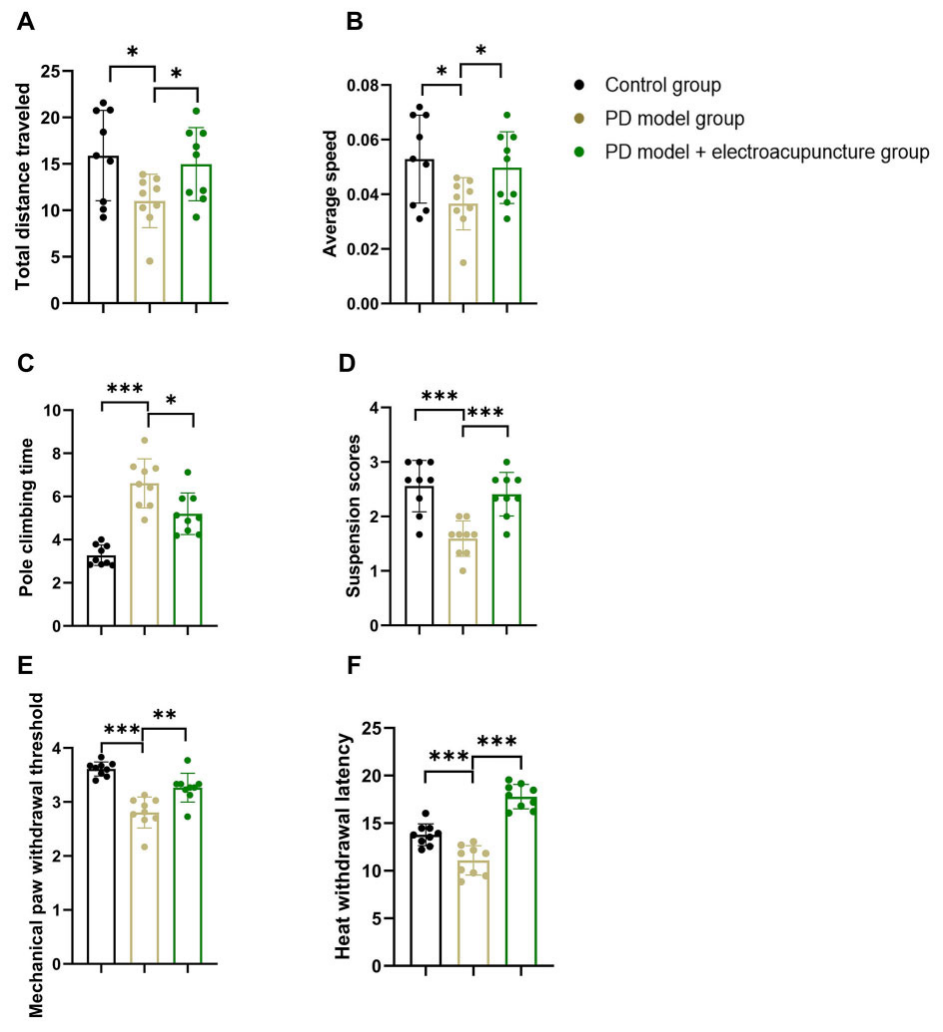
Behavior tests of motor function and nociceptive sensitivity in control, Parkinson's disease (PD), and electroacupuncture (EA)-treated PD mice. **A** and **B**, Total distance traveled (m) and mean speed (m/s) in the open field test. **C**, Pole climbing time (s) in the pole test. **D**, Suspension test scores. **E** and **F**, Mechanical paw withdrawal threshold (s) and thermal withdrawal latency (s). Data are reported as means±SE. *P<0.05, **P<0.01, ***P<0.001 (one-way ANOVA).

EA significantly improved these motor impairments. Compared to untreated PD mice, EA-treated mice exhibited increased total distance traveled (11.00±2.87 m *vs* 14.97±3.71 m, *t*=2.442, P=0.027) and average speed (0.04±0.01 m/s *vs* 0.05±0.01 m/s, *t*=2.443, P=0.027), shortened pole climbing time (6.61±1.14 s *vs* 5.20±0.91 s, *t*=−2.848, P=0.012), and elevated suspension scores (1.59±0.31 *vs* 2.41±0.38, *t*=4.734, P<0.0001) (Figure 1A-D). These findings confirmed the successful induction of motor deficits by MPTP and demonstrated that EA significantly improved locomotor function in this PD mouse model.

### EA alleviated nociceptive hypersensitivity in MPTP-induced mice

PD mice displayed pronounced nociceptive hypersensitivity, evidenced by significantly reduced mechanical paw withdrawal thresholds (3.61±0.12 s *vs* 2.81±0.27 s, *t*=7.699, P<0.0001) and shortened heat withdrawal latencies (14.03±1.27 s *vs* 11.08±1.45 s, *t*=4.317, P=0.001), compared to the control mice. EA reversed these pain-related alterations. EA-treated mice showed increased mechanical withdrawal thresholds (2.81±0.27 s *vs* 3.27±0.25 s, *t*=3.528, P=0.003) and prolonged heat withdrawal latencies (11.08±1.45 s *vs* 17.79±1.20 s, *t*=10.044, P<0.0001) (Figure 1E and F). These results indicated that EA effectively attenuated both mechanical and thermal pain hypersensitivity in PD.

### EA exerted neuroprotective effects on dopaminergic neurons

**Figure 2:**
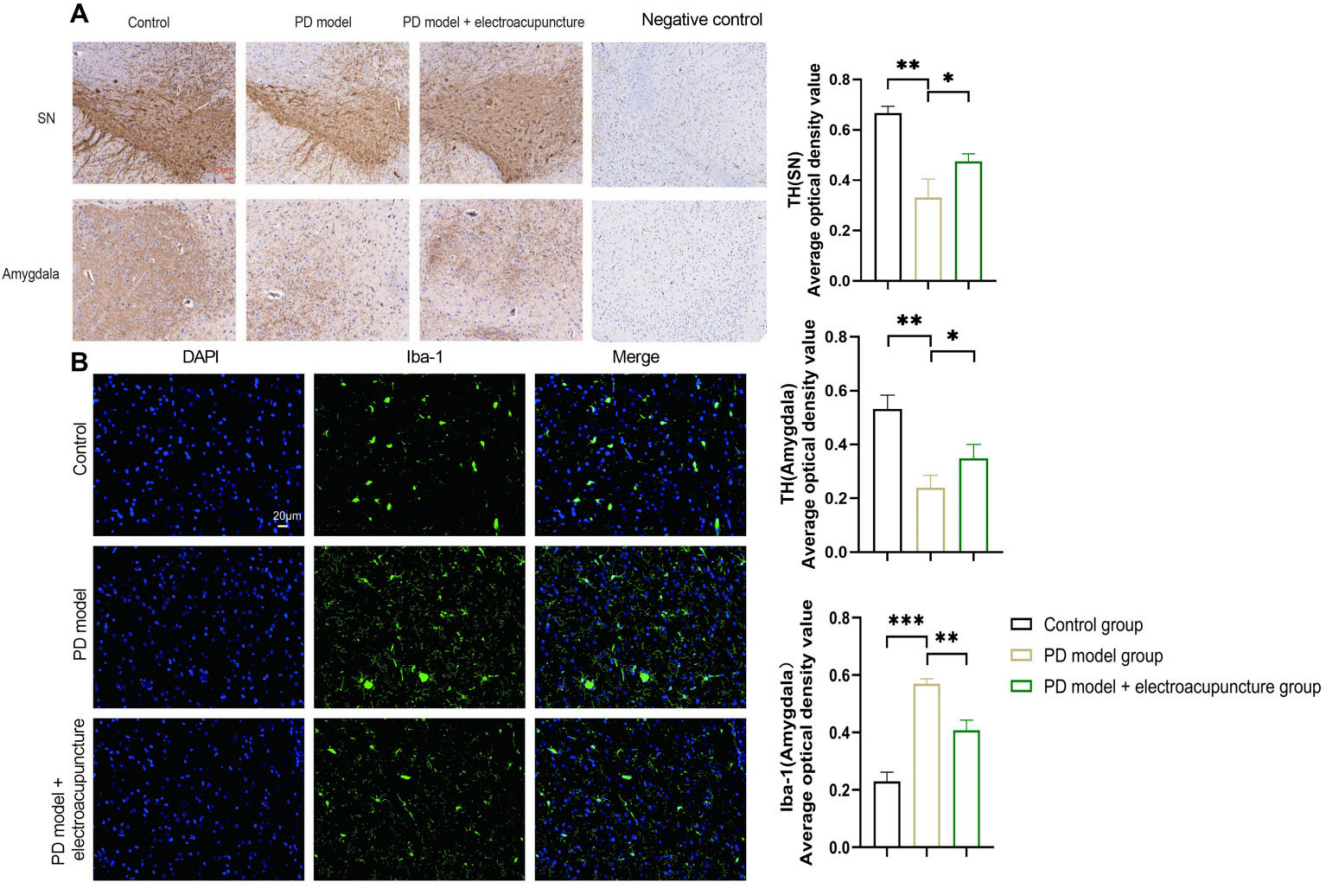
Immunohistochemical analysis of tyrosine hydroxylase (TH) and Iba1 expression in the substantia nigra (SN) and amygdala of control, Parkinson's disease (PD), and electroacupuncture (EA)-treated PD mice. **A**, Representative TH staining in the SN and amygdala (scale bar=50 μm). **B**, Representative Iba1 staining in the amygdala (scale bar=20 μm). Data are reported as means±SE. *P<0.05, **P<0.01, ***P<0.001 (one-way ANOVA).

PD mice exhibited a significant reduction in TH-positive neurons in both the SN (0.667±0.022 *vs* 0.331±0.060, *t*=7.432, P=0.002) and the amygdala (0.532±0.042 *vs* 0.240±0.037, *t*=7.336, P=0.002). EA treatment significantly increased the density of TH-positive neurons in both regions compared to untreated PD mice: SN 0.331±0.060 *vs* 0.475±0.025, *t*=3.119, P=0.036 and amygdala 0.240±0.037 *vs* 0.349±0.041, *t*=2.768, P=0.050 (Figure 2A). These results suggest that EA may confer neuroprotective effects by preserving dopaminergic neurons in both the SN and amygdala under PD-related pathological conditions.

### EA suppressed PD-related microglial activation

Immunofluorescence revealed a significant increase in Iba-1-positive microglia in the amygdala of PD mice compared to controls (0.171±0.185 *vs* 0.534±0.683, *t*=−8.882, P=0.001). EA significantly reduced Iba-1 expression in the amygdala (0.534±0.683 *vs* 0.371±0.021, *t*=−3.945, P=0.017) (Figure 2B). These findings indicated that EA effectively suppressed microglial activation in the amygdala.

### EA reduced NLRP3 inflammasome-associated protein levels in the amygdala

The NLRP3 inflammasome, composed of NLRP3, ASC, and caspase-1, plays a critical role in neuroinflammatory signaling. WB analysis revealed that expression levels of all three components were significantly elevated in the amygdala of MPTP-induced mice compared to controls (NLRP3: 0.131±0.048 *vs* 0.648±0.079, *t*=−9.669, P=0.001; ASC: 0.173±0.056 *vs* 0.735±0.099, *t*=−8.541, P=0.001; caspase-1: 0.120±0.024 *vs* 0.542±0.074, *t*=−9.355, P=0.001).

**Figure 3:**
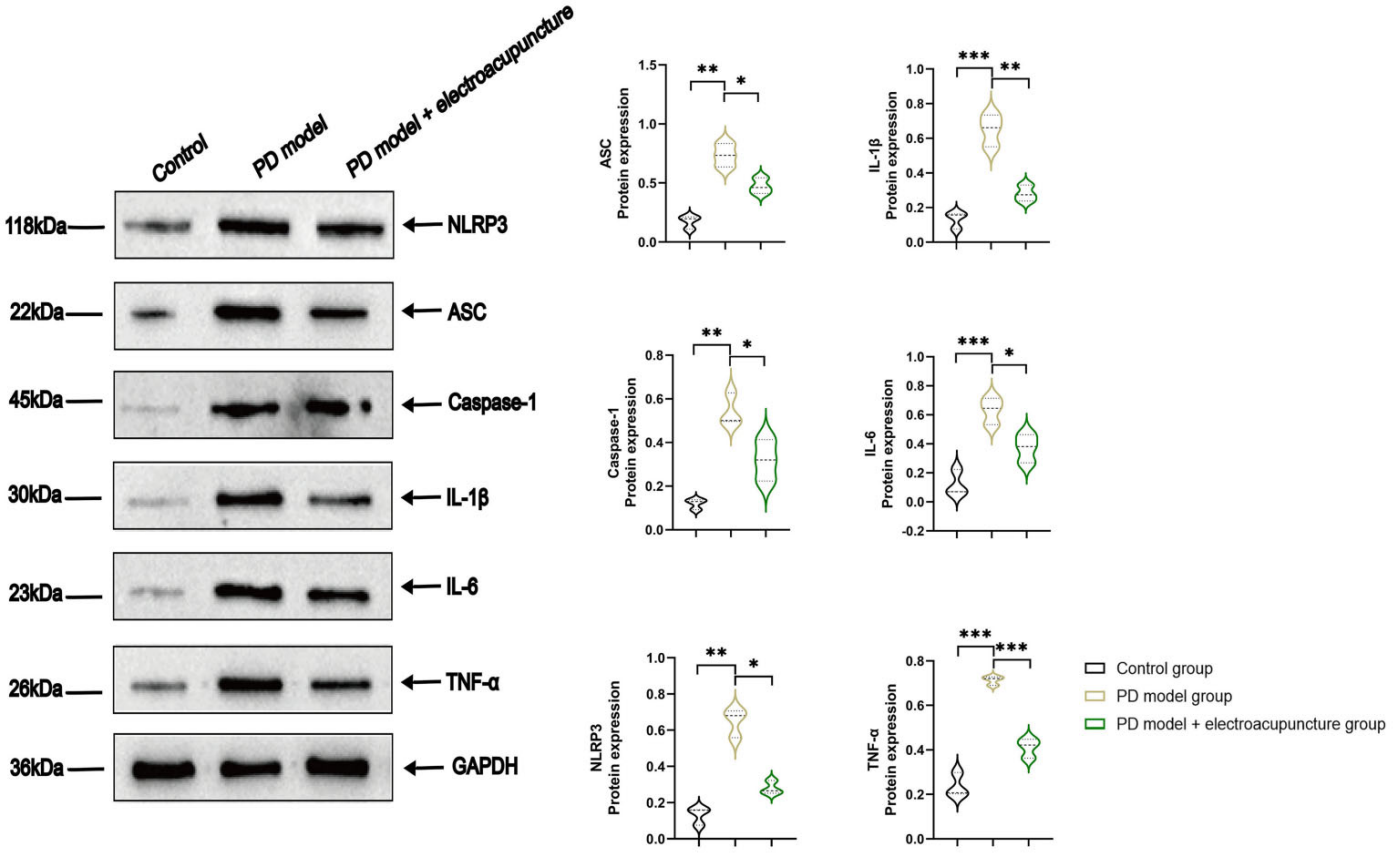
Western blot analysis of inflammasome-related and proinflammatory proteins in the amygdala of control, Parkinson's disease (PD), and electroacupuncture (EA)-treated PD mice. Representative blots and quantitative analysis of NLRP3, ASC, caspase-1, interleukin (IL)-1β, IL-6, and tumor necrosis factor (TNF)-α protein expression across the three groups. GAPDH was used as a loading control. Data are reported as means±SE. *P<0.05, **P<0.01, ***P<0.001 (one-way ANOVA).

EA treatment significantly reduced the expression of these proteins compared to the PD model group (NLRP3: 0.648±0.079 *vs* 0.280±0.037, *t*=−7.329, P=0.002; ASC: 0.735±0.099 *vs* 0.473±0.067, *t*=−3.783, P=0.019; caspase-1: 0.542±0.074 *vs* 0.319±0.095, *t*=−3.200, P=0.033) (Figure 3). These results indicated that EA effectively suppressed NLRP3 inflammasome activation in the amygdala under PD-related inflammatory conditions.

### EA attenuated pro-inflammatory cytokine expression in the amygdala

To further evaluate the neuroinflammatory response, WB was performed to measure levels of IL-1β, IL-6, and TNF-α in the amygdala. PD mice exhibited significantly higher cytokine levels compared to controls (IL-1β: 0.132±0.048 *vs* 0.649±0.092, *t*=−8.600, P=0.001; IL-6: 0.122±0.088 *vs* 0.631±0.092, *t*=−6.930, P=0.002; TNF-α: 0.236±0.054 *vs* 0.713±0.020, *t*=−14.325, P<0.0001).

EA treatment significantly decreased the expression of these cytokines relative to the untreated PD group (IL-1β: 0.649±0.092 *vs* 0.281±0.047, *t*=−6.182, P=0.003; IL-6: 0.631±0.092 *vs* 0.372±0.097, *t*=−3.349, P=0.029; TNF-α: 0.713±0.020 *vs* 0.411±0.043, *t*=−10.975, P<0.0001, Figure 3). These findings further supported the notion that EA alleviated neuroinflammation by downregulating pro-inflammatory cytokine expression in the amygdala.

## Discussion

**Figure 4:**
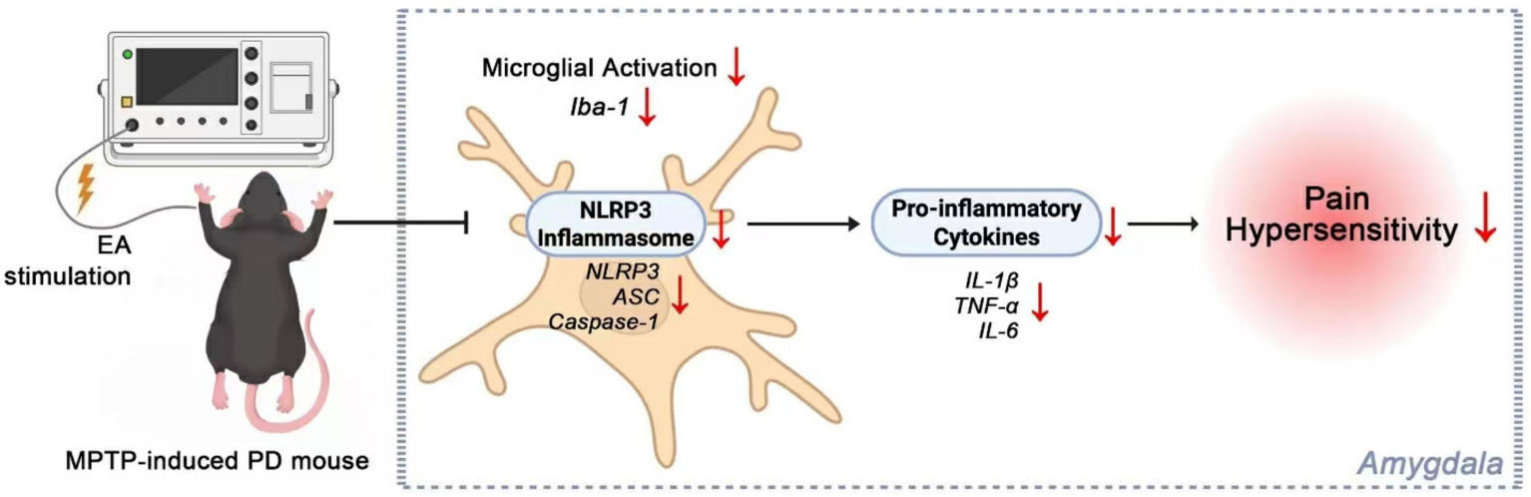
Schematic illustration of the proposed mechanism by which electroacupuncture (EA) alleviates Parkinson's disease (PD)-related pain.

This study demonstrated that EA alleviated both mechanical and thermal nociceptive hypersensitivity in a subacute MPTP-induced mouse model of PD. IHC showed that EA increased the number of TH-positive dopaminergic neurons in both the SN and amygdala. IHF indicated that EA significantly suppressed microglial activation in the amygdala. Furthermore, WB revealed that this treatment reduced amygdala expression of NLRP3 inflammasome components (NLRP3, ASC, caspase-1) and pro-inflammatory cytokines (IL-1β, IL-6, TNF-α) in the same region. Together, these findings suggest that EA exerts its analgesic effects not only through preservation of dopaminergic neurons, but also by attenuating microglial activation and subsequent inflammasome-mediated inflammatory signaling, as schematically illustrated in Figure 4. These findings offer a foundation for discussing the potential mechanisms by which EA modulates PD-related pain, particularly through dopaminergic preservation and inflammatory regulation.

Pain in Parkinson's disease (PD) frequently emerges prior to motor symptoms and is closely linked to neuroinflammatory processes that impair dopaminergic signaling (18,19). While both dopaminergic and non-dopaminergic systems contribute to PD-related pain (20), the mesolimbic dopaminergic pathway - particularly projections from the ventral tegmental area (VTA) to the amygdala, nucleus accumbens (NAc), and prefrontal cortex - plays a central role in pain modulation (21-23). Disruptions in the structure and function of this circuit have been consistently observed in individuals with chronic pain. Our findings suggest that EA may help preserve dopaminergic neurons in key mesolimbic regions involved in pain modulation.

Neuroinflammation, primarily mediated by activated microglia, is a hallmark of PD and is closely associated with its non-motor symptoms, including pain (24,25). In response to pathogen- or damage-associated molecular patterns, microglia activate pattern recognition receptors (PRRs) and release a cascade of pro-inflammatory cytokines, such as TNF-α, IL-1β, and IL-6 (26-28). In our study, MPTP-induced mice exhibited increased Iba-1-positive microglia in the amygdala, which were significantly reduced following EA treatment. These findings suggest that EA suppresses microglial activation, which may disrupt the pro-inflammatory cascade contributing to PD-related pain hypersensitivity. This is consistent with previous studies showing that dopamine D2 receptor signaling in microglia exacerbates inflammation (29), and that α-synuclein aggregates further amplify microglial reactivity via TLR2 and TLR5 pathways (30,31). Moreover, microglial activation has been shown to promote peripheral pain hypersensitivity through P2X4 and CCR1-dependent mechanisms (32,33), highlighting the critical role of microglial modulation in both central and peripheral pain regulation.

Downstream of microglial activation, the NLRP3 inflammasome plays a central role in PD-associated neuroinflammation. Primarily expressed in microglia, the NLRP3 inflammasome is essential for the brain's immune response to injury and disease (34). This multiprotein complex - comprising NLRP3, ASC, and caspase-1 - drives the maturation and secretion of pro-inflammatory cytokines, notably IL-1β and IL-18 (35,36). Inhibition of the NLRP3 inflammasome has been shown to attenuate PD-related neurotoxicity and inflammation in previous studies (37,38). In line with these findings, our results demonstrated that EA significantly downregulated the expression of NLRP3 inflammasome components in the amygdala of MPTP-induced mice, suggesting that this pathway may be a key therapeutic target of EA.

EA has been widely used in the treatment of various types of pain, including both neuropathic and nociceptive pain, and has been shown to improve quality of life in patients with PD (39). Mechanistically, EA has been reported to inhibit glial activation, promote microglial M2 polarization, and modulate inflammatory signaling (40). Experimental studies suggest that EA alleviates inflammatory pain by inhibiting CB2-dependent activation of the NLRP3 inflammasome, enhancing the expression of tyrosine hydroxylase and dopamine receptors, and downregulating pro-inflammatory cytokines such as TNF-α and IL-1β in pain-related brain regions (3). Our findings are consistent with these reports, demonstrating that EA suppressed microglial activity and NLRP3-mediated cytokine production in the amygdala. These results suggest that EA may exert its analgesic effects, at least in part, by modulating central neuroimmune pathways.

### Study limitations

Several limitations of this study must be acknowledged. First, although the MPTP-induced model is widely used, it does not replicate the α-synuclein pathology characteristic of human PD. Second, we did not include a healthy EA-only or sham EA control group, nor a pharmacological comparator such as L-DOPA, which may limit interpretation regarding disease specificity, stimulation dependency, and comparative efficacy. Third, although the EA needle positioning was guided by stereotaxic coordinates, no physiological confirmation (e.g., electrophysiological mapping) was performed. Fourth, we focused exclusively on the amygdala due to its key role in pain and neuroinflammation but did not examine other pain-relevant regions such as the periaqueductal gray (PAG) and anterior cingulate cortex (ACC), which are also implicated in PD-related pain modulation and affective processing, and are often assessed through behavioral paradigms beyond nociceptive thresholds. Finally, while we observed changes in NLRP3 inflammasome markers, we did not include causal validation such as NLRP3 inhibition or knockout models, limiting mechanistic interpretation. Future studies should incorporate α-synuclein models, pharmacological controls, cellular colocalization analysis, high-resolution morphological assessments, and broader pain-processing circuitry analysis.

## Conclusion

The present study provided evidence that EA mitigated PD-associated pain by modulating microglial activation and inhibiting NLRP3 inflammasome signaling in the amygdala. These findings highlight the therapeutic potential of EA as a neuromodulator strategy targeting neuroimmune pathways in PD-related pain.

## Data Availability

The data that support the findings of this study are available from the corresponding author upon reasonable request.
